# Gui-A-Gra Attenuates Testicular Dysfunction in Varicocele-Induced Rats via Oxidative Stress, ER Stress and Mitochondrial Apoptosis Pathway

**DOI:** 10.3390/ijms21239231

**Published:** 2020-12-03

**Authors:** Keshab Kumar Karna, Na Young Choi, Chul Young Kim, Hye Kyung Kim, Yu Seob Shin, Jong Kwan Park

**Affiliations:** 1 Department of Urology, Institute for Medical Sciences, Jeonbuk National University Medical School, Jeonju 54907, Korea; karnakeshab@gmail.com (K.K.K.); cybernyy@naver.com (N.Y.C.); 2 Biomedical Research Institute and Clinical Trial Center for Medical Device, Jeonbuk National University Hospital, Jeonju 54907, Korea; 3 College of Pharmacy, Hanyang University, Ansan 426791, Korea; chulykim@hanyang.ac.kr; 4 College of Pharmacy, Kyungsung University, Busan 48434, Korea; fiona30@ks.ac

**Keywords:** Gui-A-Gra, apoptosis, oxidative stress, endoplasmic reticulum (ER) stress, inflammation, mitochondria, testis, steroidogenic acute regulatory (StAR) protein, varicocele

## Abstract

Gui-A-Gra, a commercial insect powder from *Gryllus bimaculatus*, is registered as an edible insect by the Korean food and drug administration. The aim of this study was to investigate the effect of Gui-A-Gra on testicular damage induced by experimental left varicocele in male Sprague Dawley rats. A total of 72 rats were randomly divided into the following six groups (12 rats in each group): a normal control group (CTR), a group administrated with Gui-A-Gra 1.63 gm/kg (G1.63), a group administrated with Gui-A-Gra 6.5 gm/kg (G6.5), a varicocele (VC)-induced control group (VC), a VC-induced group administrated with Gui-A-Gra 1.63 gm/kg (VC + G1.63), and a VC-induced group administrated with Gui-A-Gra 6.5 gm/kg (VC + G6.5). Rats were administrated 1.63 or 6.5 gm/kg Gui-A-Gra once daily for 42 days. Indicators of sperm parameters, histopathology, reproductive hormones, oxidative stress, endoplasmic reticulum (ER) stress, inflammation, and mitochondrial apoptosis were analyzed to evaluate effects of Gui-A-Gra on VC-induced testicular dysfunction. Gui-A-Gra administration to VC-induced rats significantly (*p* < 0.05) increased sperm count and sperm motility, Johnsen score, spermatogenic cell density, serum testosterone, testicular superoxide dismutase (SOD), glutathione peroxidase (GPx), catalase, GPx4, and steroidogenic acute regulatory protein (StAR) level. Moreover, pretreatment with Gui-A-Gra significantly (*p* < 0.05) decreased terminal deoxynucleotidyl transferase-mediated dUTP nick-end labeling (TUNEL) positive cells/tubules, serum luteinizing hormone (LH), serum follicle-stimulating hormone (FSH), testicular tumor necrosis factor-α (TNF-α), interleukin-6 (IL-6), malondialdehyde (MDA), reactive oxygen species (ROS)/reactive nitrogen species (RNS) level, glucose-regulated protein-78 (Grp-78), phosphorylated c-Jun-N-terminal kinase (p-JNK), phosphorylated inositol-requiring transmembrane kinase/endoribonuclease 1α (p-IRE1α), cleaved caspase-3, and BCL2 associated X protein: B-cell lymphoma 2 (Bax: Bcl2) ratio in VC rats. These results suggest that protective effects of Gui-A-Gra on VC-induced testicular injury might be due to its antioxidant, anti-inflammatory, and androgenic activities that might be mediated via crosstalk of oxidative stress, ER stress, and mitochondrial apoptosis pathway.

## 1. Introduction

Varicocele (VC) is characterized by dilation and tortuosity of internal spermatic vein (pampiniform plexus), a network of veins that drains testicular tissues [1]. VC is considered as one of major attributable causes of male infertility [2,3]. The incidence of clinical VC in male population is approximately 15%. Approximately 30–40% of men with VC have primary infertility problems and about 70–80% of men with VC have secondary infertility [1,4]. VC occurs more frequently on the left side and the etiology of VC is multifactorial [5,6]. Adverse effects of VC on male reproduction are associated with decreased testicular volume, sperm count, sperm motility, testosterone level, and Leydig cell function and increased germ cell apoptosis [7]. The pathophysiology of VC and molecular mechanisms involved in testicular damage and sperm dysfunctions have not been fully elucidated yet. It is generally believed that VC can induce an inhibition of testosterone biosynthesis, sperm chromosomal abnormalities, and elevation of reactive oxygen species (ROS) that can lead to oxidative stress and endoplasmic reticulum (ER) stress and trigger germ cell apoptosis [1,8]. VC associated infertility is correctable by surgical treatment including embolization, open scrotal or inguinal varicocelectomy, microsurgical varicocelectomy, and laparoscopic varicocelectomy [9]. Nevertheless, whether these operations can reverse altered semen parameters to baseline values in VC patients remains controversial [10]. Since there is no definite cure for VC-induced infertility, adjuvant therapies have been developed to assist varicocelectomy.

Oxidative stress and testicular apoptosis are well documented causes of testicular dysfunction [1,3]. Plasma membranes of spermatozoa have specific compositions of phospholipids and significant concentrations of saturated and unsaturated fatty acids that make them susceptible to oxidative stress caused by increased production of reactive oxygen species (ROS) [11]. At the same time, damaged germinal epithelium, abnormal spermatozoa, and apoptotic spermatogenic cells can significantly elevate ROS production in testicular tissues [12]. Under clinical VC conditions, ROS production can exceed endogenous antioxidant capacity and increased ROS can elevate oxidative stress and ER stress [1,2,3]. Prolonged ER stress in VC can initiate testicular apoptosis by activating inositol-requiring transmembrane kinase/endoribonuclease 1 (IRE 1)-c-Jun amino terminal kinase (JNK) pathway in rats [13]. The mitochondrial signaling pathway is also interlinked for testicular tissue apoptosis in VC-induced rats [14].

It has been reported that antioxidant supplements can alleviate male infertility by scavenging ROS and modulating inflammatory responses and the antioxidant system [15]. Gui-A-Gra is a commercial *Gryllus bimarculatus* powder that has recently been registered as an edible insect in Korea [16]. *Gryllus bimarculatus* has pharmacological activity such as antioxidant and anti-inflammatory effects [17,18]. A recent study has shown that chitin and chitosan compound obtained from the exoskeletons of *Gryllus bimarculatus* have beneficial antioxidant, hypocholesterolemic, and antimicrobial properties [19]. The constituents of *G. bimaculatus* include glycosaminoglycan which exhibits anti-inflammatory effects [20]. Recent reports using animal models have shown that *Gryllus bimarculatus* extract can prevent diabetes, liver disease, and arthritis [21,22,23]. However, the effect of *Gryllus bimarculatus* on male reproductive functions has not been reported yet. Therefore, the objective of this study was to determine the protective effect of *Gryllus bimaculatus* powder for the recovery from testicular dysfunction and its associated male fertility condition in a VC-induced rat model. The roles of inflammation, oxidative stress, endoplasmic reticulum (ER) stress, and mitochondrial (intrinsic) pathway in VC testicular tissues were also examined to determine molecular mechanisms involved in the protective effect of *Gryllus bimaculatus*.

## 2. Results

### 2.1. Body Weight, Organ Weights, Fertility Parameters, Sperm Count, and Sperm Motility

Effects of GUI-A-GRA on body weight, organ weights, sperm count, and sperm motility in both vas deferens and epididymis are summarized in Table 1 and Table 2. Final body and organ weights were similar among all groups (Table 1). Sperm count and sperm motility in both vas deference and epididymis were decreased in the VC group as compared to the control (CTR) group, although such decreases were not statistically significant except for the decrease of sperm count in vas deference which was significant (*p* < 0.05). Pretreatment with G 6.5 significantly increased sperm count in both vas deference and epididymis of VC rats (*p* < 0.05). Sperm motility in vas deference and epididymis in VC rats showed remarkable increases after pretreatment with G1.63 or G6.5 (*p* < 0.05) (Table 2). The fertility rate and pups per female were presented in Table S1. The fertility rate and pups per female were decreased in the VC group as compared to the control (CTR) group, although such decreases were not statistically significant. However, the pup per female in VC + G6.5 group was significant increased as compared to the VC group (Table S1).

### 2.2. Gui-A-Gra Treatment Counteracts Damage to Seminiferous Tubules

**Figure 1:**
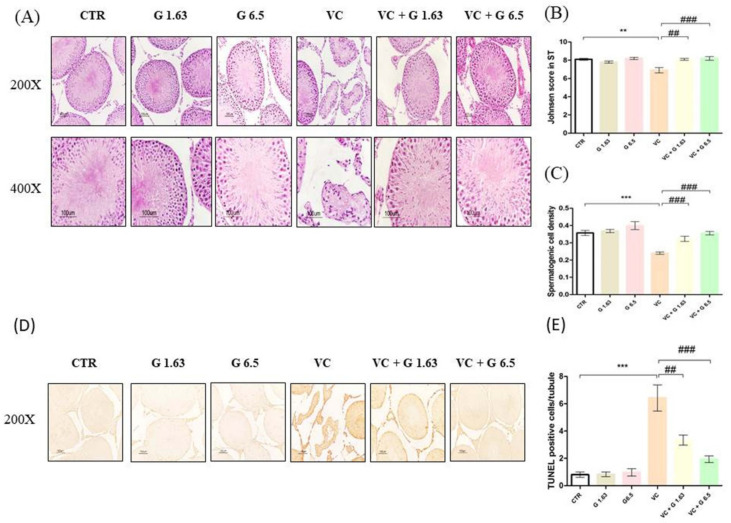
Histopathological morphology changes in rat left testis by H&E staining and germ cell apoptosis by TUNEL staining. (**A**) Photomicrogram of rat testis showing irregular seminiferous tubules (STs), narrow germinal cell layer, and absence of spermatozoa in the VC group. (**B**) Johnsen’s score in testis tissues. (**C**) Spermatogenic cell density in testis tissues. (**D**) Apoptotic testis principle cells after TUNEL staining. (**E**) The number of TUNEL positive cells per seminiferous tubule. Data are presented in mean ± S.E.M, *n* = 12. Statistical analyses were performed using one-way ANOVA followed by Tukey’s post-hoc test. ** *p* < 0.01 and *** *p* < 0.001 vs. CTR group; ^##^ *p* < 0.01 and ^###^ *p* < 0.001 vs. VC group. CTR, control; G1.63, Gui-A-Gra 1.63 gm/kg p.o.; G6.5, Gui-A-Gra 6.5 gm/kg p.o.; VC, varicocele; VC + G1.63, varicocele + Gui-A-Gra 1.63 gm/kg p.o.; VC + G6.5, varicocele + Gui-A-Gra 6.5 gm/kg p.o.; G, Gui-A-Gra; p.o., per oral; H&E, hematoxylin and eosin; TUNEL, terminal deoxynucleotidyl transferase-mediated dUTP nick-end labeling; ANOVA, analysis of variance; SEM: standard error of the mean.

Histological analysis of hematoxylin and eosin staining in testis showed normal morphology with normal numbers of mature sperm in seminiferous tubules of all sham groups. However, fewer cell layers, absence of sperm, atrophy, and vacuolation were observed in the VC group (Figure 1A). More germ cell apoptosis (Figure 1D) and higher numbers of TUNEL positive cells/tubules (Figure 1E) were also detected in the VC group as compared to the CTR group (*p* < 0.05). Johnsen’s score and spermatogenic cell density in the VC group were significantly (*p* < 0.05) decreased compared to those in the CTR group (Figure 1B,C). They were effectively reverted in Gui-A-Gra pretreated groups (VC+ G1.63 group and VC+ G6.5 group) (Figure 1A–E). No significant difference was observed between VC + G1.63 and VC + G6.5 group.

### 2.3. Reproductive Hormone Levels and Inflammatory Markers

**Figure 2:**
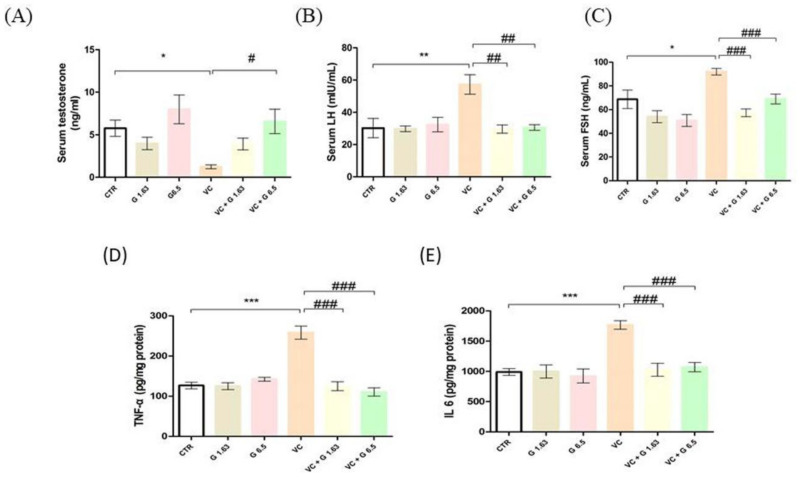
Effect of Gui-A-Gra on serum hormone levels and inflammatory cytokines levels in VC-induced male SD rats. (**A**) Serum testosterone, (**B**) serum LH, (**C**) serum FSH, (**D**) testicular TNF-α, (**E**) testicular IL-6. Data are presented in mean ± S.E.M, *n* = 12. Statistical analyses were performed using one-way ANOVA followed by Tukey’s post-hoc test. * *p* < 0.05; ** *p* < 0.01; and *** *p* < 0.001 vs. CTR group; ^#^ *p* < 0.05; ^##^ *p* < 0.01; and ^###^ *p* < 0.001 vs. VC group. CTR, control; G1.63, Gui-A-Gra 1.63 gm/kg p.o.; G6.5, Gui-A-Gra 6.5 gm/kg p.o.; VC, varicocele; VC + G1.63, varicocele + Gui-A-Gra 1.63 gm/kg p.o.; VC + G6.5, varicocele + Gui-A-Gra 6.5 gm/kg p.o.; G, Gui-A-Gra; LH, luteinizing hormone; FSH, follicle stimulating hormone; IL-6, interleukin-6; TNF-α, tumor necrosis factor-α; p.o., per oral; ANOVA, analysis of variance; SEM: standard error of the mean.

Serum testosterone level was significantly decreased in the VC group as compared to the CTR group (Figure 2). It was significantly increased in the Gui-A-Gra + VC group as compared to the VC group. Serum LH and FSH levels in the VC group were remarkably increased (*p* < 0.05) as compared to the CTR group. However, serum LH and FSH levels were significantly decreased in VC + G1.63 and VC + G6.5 groups. Levels of IL-6 and TNF-α were remarkably (*p* < 0.05) upregulated in the VC group as compared to the CTR group. Pretreatment with Gui-A-Gra significantly (*p* < 0.05) downregulated these parameters in the VC + G1.63 and VC + G6.5 groups (Figure 2).

### 2.4. Determination of Testicular Lipid Peroxidation and Antioxidant Enzyme Activities

**Figure 3:**
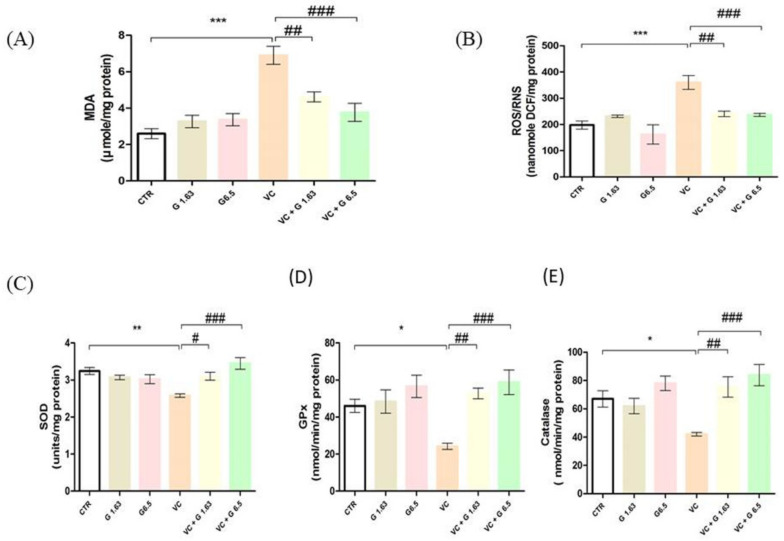
Effect of Gui-A-Gra treatment on testicular oxidative stress biomarkers in VC-induced male Sprague Dawley rats. (**A**) Malondialdehyde level (MD), (**B**) ROS/RNS level, (**C**) SOD activity, (**D**) GPx level, (**E**) catalase level. Data are presented in mean ± S.E.M, *n* = 12. Statistical analyses were performed using one-way ANOVA followed by Tukey’s post-hoc test. * *p* < 0.05; ** *p* < 0.01; and *** *p* < 0.001 vs. CTR group; ^#^ *p* < 0.05; ^##^ *p* < 0.01; and ^###^ *p* < 0.001 vs. VC group. CTR, control; G1.63, Gui-A-Gra 1.63 gm/kg p.o.; G6.5, Gui-A-Gra 6.5 gm/kg p.o.; VC, varicocele; VC + G1.63, varicocele + Gui-A-Gra 1.63 gm/kg p.o.; VC + G6.5, varicocele + Gui-A-Gra 6.5 gm/kg p.o.; G, Gui-A-Gra; ROS/RNS, reactive oxygen species/ reactive nitrogen species; SOD, superoxide dismutase; GPx, glutathione peroxidase; p.o., per oral; ANOVA, analysis of variance; SEM: standard error of the mean.

Results of lipid peroxidation parameters (malondialdehyde (MDA), ROS/RNS) and antioxidant enzymes (SOD, GPx, and catalase) in testicular tissues are presented in Figure 3. VC-induced rats showed a significantly (*p* < 0.05) enhanced MDA level and ROS/RNS level compared to rats in the CTR group. Treatment with G1.63 and G6.5 significantly (*p* < 0.05) restored these parameters in the VC group. VC-induced rats showed significant decreases of testicular levels of SOD, GPx, and catalase levels as compared to rats in the CTR group. These levels were remarkably (*p* < 0.05) increased after pre-treatment with G1.63 or G6.5. No significant difference in these parameters was observed between VC + G1.63 and VC + G6.5 groups.

### 2.5. Western Blot and Immunohistochemistry of Proteins Expressed in Left Testis

**Figure 4:**
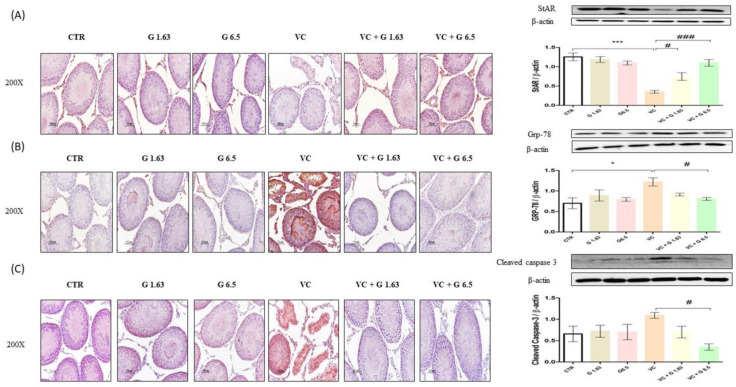
Effect of Gui-A-Gra on protein expression levels of StAR, Grp-78 and cleaved caspase-3 in testis tissues of VC-induced male Sprague Dawley rats determined by immunohistochemistry staining and western blot analysis. (**A**) StAR, (**B**) Grp-78, (**C**) cleaved caspase-3. Statistical analyses were performed using one-way ANOVA followed by Tukey’s post-hoc test. * *p* < 0.05 and *** *p* < 0.001 vs. CTR group; ^#^ *p* < 0.05 and ^###^ *p* < 0.001 vs. VC group. CTR, control; G 1.63, Gui-A-Gra 1.63 gm/kg p.o.; G6.5, Gui-A-Gra 6.5 gm/kg p.o.; VC, varicocele; VC + G1.63, varicocele + Gui-A-Gra 1.63 gm/kg p.o.; VC + G6.5, varicocele + Gui-A-Gra 6.5 gm/kg p.o.; G, Gui-A-Gra; StAR, steroidogenic acute regulatory protein; Grp-78, glucose regulated protein-78; p.o., per oral; ANOVA, analysis of variance; SEM: standard error of the mean.

**Figure 5:**
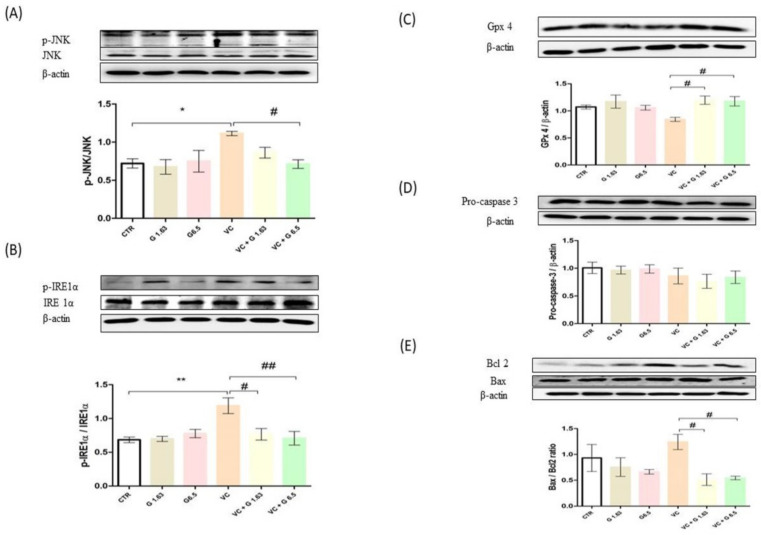
Effect of Gui-A-Gra on protein expression levels of JNK, p-JNK, IRE 1α, p-IRE 1α, Gpx4, pro-caspase 3, and Bax/Bcl2 ratio in testis tissues of VC-induced male Sprague Dawley rats determined by western blot analysis. (**A**) p-JNK/JNK, (**B**) p-IRE1α/IRE1α, (**C**) GPx4, (**D**) pro-caspase-3, and (**E**) Bax/Bcl2 ratio. Data are presented in mean ± S.E.M, *n* = 12. Statistical analyses were performed using one-way ANOVA followed by Tukey’s post-hoc test. * *p* < 0.05 and ** *p* < 0.01 vs. CTR group; ^#^ *p* < 0.05 and ^##^ *p* < 0.01 vs. VC group. CTR, control; G 1.63, Gui-A-Gra 1.63 gm/kg p.o.; G6.5, Gui-A-Gra 6.5 gm/kg p.o.; VC, varicocele; VC + G1.63, varicocele + Gui-A-Gra 1.63 gm/kg p.o.; VC + G6.5, varicocele + Gui-A-Gra 6.5 gm/kg p.o.; G, Gui-A-Gra; p-JNK/JNK, phosphorylated c-jun-N-terminal kinase/c-jun-N-terminal kinase; p-IRE1α/IRE1α, phosphorylated inositol-requiring transmembrane kinase 1α/ inositol-requiring transmembrane kinase 1α ; GPx4, glutathione peroxidase-4; Bax;Bcl2, BCL2 associated X protein: B-cell lymphoma 2; p.o., per oral; ANOVA, analysis of variance; SEM: standard error of the mean.

To analyze the protective mechanism of Gui-A-Gra against VC-induced testicular dysfunction, protein expression levels of Grp 78, p-JNK/JNK, p-IRE1α/IRE1α caspase 3, cleaved caspase-3, BCL2 associated X protein (Bax): B-cell lymphoma 2 (Bcl2) ratio, StAR, and Gpx4 were analyzed in testis tissues by western blotting. Results are shown in Figure 4 and Figure 5. VC-induced rats showed significant (*p* < 0.05) increases of GRP-78, p-IRE1α/ IRE1α, and p-JNK/JNK as compared to rats in the CTR group. In contrast, levels of GRP-78, p-IRE1α/IRE1α, and p-JNK/JNK levels in VC + G6.5 group were remarkably (*p* < 0.05) decreased as compared to those in the VC group. Levels of GRP 78 and p-JNK/JNK in VC + G1.63 group were lower than in the VC group, although differences between the two groups were not statistically significant. However, the p-IRE1α/IRE1α level in VC + G1.63 group was significant decreased as compared to the VC group. Levels of pro-caspase-3 were similar among all groups. VC-induced rats showed prominently increased expression of cleaved caspase-3 and Bax:Bcl2 ratio compared to rats in the CTR group, although the increase was not statistically significant compared with the CTR group. In contrast, the cleaved caspase 3 and Bax:Bcl2 ratio in the VC + G6.5 group was significantly (*p* < 0.05) decreased as compared to the VC group (Figure 4C and Figure 5E). The cleaved caspase-3 level in the VC + G1.63 group was decreased as compared to that in the VC group. However, this decrease was not statistically significant. On the other hand, the Bax: Bcl2 ratio in the VC + G1.63 was significantly decreased compared to that in the VC group.

VC-induced rats showed significantly decreased expression levels of StAR as compared to rats in the CTR group. However, treatment with Gui-A-Gra restored levels of StAR in varicocelized rats. Cell-specific expression of StAR, Grp 78, and cleaved caspase 3 proteins in testis tissues was further analyzed by immunohistochemistry staining (Figure 4A–C). A dark red positive signal of StAR protein was observed in the Leydig cells of the CTR group, the G1.63 group, and the G6.5 group. However, the StAR protein in the VC group showed faint expression. In contrast, pretreatment with Gui-A-Gra enhanced StAR protein expression (dark red signal) in the Leydig cells of VC-induced rats. A dark brown positive signal of Grp 78 was observed in the VC group. In contrast, no signal of Grp 78 was observed in other groups. Similarly, deep brown staining showing positive signal of cleaved caspase 3 was detected in the VC group whereas no such signal was detected in other groups.

## 3. Discussion

The present work used an experimental VC model to determine treatment effect of Gui-A-Gra on VC. This work extended studies on pathophysiological mechanisms associated with VC and suggested an ameliorative effect of Gui-A-Gra on VC. This study is first to report a protective effect of Gui-A-Gra on testicular dysfunction and male infertility using a VC-induced rat model. Pretreatment with Gui-A-Gra significantly improved sperm parameters, regulated levels of reproductive hormones, and promoted spermatogenesis in VC rats. Results of testicular morphological analysis, TUNEL assay, immunohistochemistry, and western blot analyses indicated that Gui-A-Gra could also decrease germ cell apoptosis.

Previous studies have shown testicular hypotrophy in patients with clinical VC, but not in patients with subclinical VC [24]. In the present study, there was no significant difference in body weight or reproductive organ weight among all groups. Testicular weight in the VC group showed a decrease compared to that in the CTR group. However, such decrease was not significant. VC is considered one of the most common causes of decreased quality of sperm parameters in adult patients and experimental rat models [2,25]. Similar results were found in VC rats of the present study. Decreased quality of sperm parameters might be due to increased ROS level and decreased activities of antioxidant enzymes, leading to sperm apoptosis [26]. Pretreatment with Gui-A-Gra improved sperm count and sperm motility of vas deference and epididymis in VC rats. Increased sperm counts were due to a curative effect of Gui-A-Gra on VC-induced testicular dysfunction in relation to spermatogenesis.

In our study, VC-induced rats showed degenerative changes in mammalian testis such as testicular atrophy, apoptosis of germ cell, aberration of STs, and impaired spermatogenesis. Similar findings have been reported in previous experimental VC studies [1,2,3]. Testosterone plays an essential role in the development of STs. Aberration of STs might be due to downregulation of testosterone biosynthesis [2]. Pretreatment with Gui-A-Gra can prevent testicular atrophy, sloughing of germ cells, and apoptosis in VC rats. Johnsen score and spermatogenic cell density are histological prognostic indicators for evaluating testicular tissues. Numerous studies have shown downregulation of these indicators in VC rats [27,28]. Consistent with previous findings, the current study also showed that the Johnsen’s score and spermatogenic cell density in STs were decreased in VC-induced rats. Administration of Gui-A-Gra remarkably up-regulated these indicators. Thus, Gui-A-Gra might be used as a supplementary therapeutic option to improve VC-induced infertility.

Leydig cells are located in the interstitial tissue of the testis where testosterone is synthesized. They are essential for spermatogenesis [29]. The StAR protein is a rate-limiting step in steroidogenesis. It plays an essential role in transferring cholesterol from the outer mitochondrial membrane to the inner mitochondrial membrane where cytochrome P450 cholesterol side chain cleavage (P450ssc) enzyme cleaves cholesterol to pregnenolone which is further converted to testosterone and various steroid hormones with a series of enzymes [30]. In the present study, aggravation of VC was associated with decreased serum testosterone level and expression of StAR protein with upregulation of serum LH and FSH levels. Spermatogenesis is regulated by the hypothalamic-pituitary-testicular axis system [31]. Upregulation of serum LH and FSH levels might be due to a feedback mechanism of the hypothalamus-pituitary-testicular axis [32]. Pretreatment with Gui-A-Gra increased StAR protein expression and serum testosterone level but downregulated serum LH and FSH levels, suggesting androgenic properties of Gui-A-Gra.

In the present study, higher concentrations of testicular MDA were detected in VC rats. The results of our study were similar to the MDA results of human testicular biopsy samples from infertile patients with VC [33]. Furthermore, increased ROS/RNS levels and decreased antioxidant enzymes such as SOD, GPx, and catalase were found in VC rats. Similar results were reported for seminal and plasma specimens of infertile patients with VC [34]. The cell membrane of spermatozoa is predominantly made of saturated and unsaturated fatty acids. Thus, it is especially prone to ROS-induced damage mediated by the formation of MDA [35]. ROS is positively correlated with degenerative changes in testicular function such as damaged DNA and protein and germ cell apoptosis [3,36]. Pretreatment with Gui-A-Gra upregulated antioxidant enzymes levels but downregulated ROS levels in VC rats, suggesting that Gui-A-Gra could decrease lipid peroxidation in testis tissues due to its antioxidant activity. Our results also revealed that Gui-A-Gra could decrease levels of inflammatory cytokines in testis tissues of VC rats. Increased levels of pro-inflammatory cytokines and decreased antioxidant capacity are associated with infertility in VC patients [37]. Inflammatory mediators are known to play a regulatory role in spermatogenic cell development, functions of sertoli cells, and sperm maturation [38]. The present study confirms that Gui-A-Gra possesses anti-inflammatory activity, consistent with a previous report [20].

**Figure 6:**
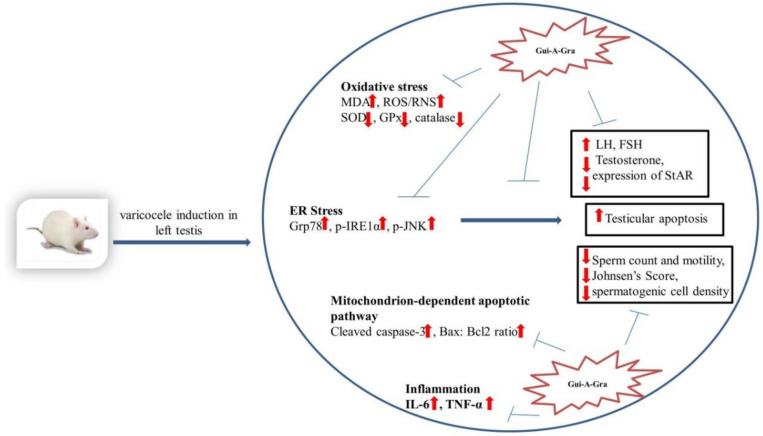
Schematic diagram showing how Gui-A-Gra can prevent testicular dysfunction in experimental varicocele (VC) rats. MDA, malondialdehyde; ROS/RNS, reactive oxygen species/reactive nitrogen species; SOD, superoxide dismutase; GPx, glutathione peroxidase; ER, endoplasmic reticulum; GRP-78, glucose-regulated protein-78; p-JNK, phosphorylated c-Jun-N-terminal kinase; p-IRE1α, phosphorylated inositol-requiring transmembrane kinase/endoribonuclease 1α; Bax:Bcl2, BCL 2 associated X protein : B-cell lymphoma 2; IL-6, interleukin-6; TNF-α, tumor necrosis factor-α; LH, luteinizing hormone; FSH, follicle stimulating hormone; StAR: steroidogenic acute regulatory protein.

Apoptosis is another detrimental factor involved in the impairment of sperm quality in patients with VC [39]. Increased levels of ROS in VC could result in peroxidation of the cell membrane of spermatozoa, resulting in apoptosis of sperm cells [34]. ROS play a bidirectional role in oxidative stress and ER stress [40,41,42]. The present study showed that ER stress, oxidative stress, and mitochondrial apoptosis pathway were key mechanisms involved in germ cell apoptosis of testis tissues in experimental VC rats. The results of our study were consistent with previous findings [1,2,13]. Upon prolonged ER stress, PERK and IRE1 mediated stress signaling can trigger apoptosis of cells [43,44]. Several studies have indicated that cleaved caspase 3 and Bax are upregulated whereas Bcl2 is downregulated in VC, which in turn can decrease the activity of antioxidant enzymes and increase tissue levels of MDA [1,2]. Bax protein is negatively correlated with the number of spermatozoa, sperm morphology, and sperm motility, whereas Bcl-2 exhibits a significant positive association with semen parameters [2,13]. In the present study, pretreatment with Gui-A-Gra significantly decreased oxidative stress, IRE1-JNK signaling, and mitochondrial (intrinsic) signaling mediated apoptosis pathways, suggesting that Gui-A-Gra could promote spermatogenesis by downregulating germ cell apoptosis (Figure 6).

## 4. Materials and Methods

### 4.1. Gui-A-Gra Material and Extract Preparation

A commercial *Gryllus bimarculatus* powder material under the name of Gui-A-Gra used for the experiments was supplied from 239bio Inc. (Iksan, Chonbuk, South Korea), a facility accredited for hazard analysis and critical control point (HACCP) and good manufacturing practice (GMP). Cultivated adult *G. bimarculatus* were subjected to 1-day of defecation before sacrifice. The *G. bimarculatus* were then cleansed with tap water three times, sterilized at 100 °C for 3–5 min, and dried for 6 h to have moisture less than 5%. Finally, dried *G. bimaculatus* were ground and homogenized. The powder was then stored at −20 °C for further use.

### 4.2. Animal Care and Experimental Design

Seventy-two 7–8 weeks old male Sprague Dawley rats were purchased from Koatech (Gyeonggi-do, South Korea) and randomly divided into six groups (12 rats/group): (1) normal control (CTR) group; (2) Gui-A-Gra 1.63 gm/kg p.o. group (G1.63); (3) Gui-A-Gra 6.5 gm/kg p.o. group (G6.5); (4) varicocele (VC) group; (5) VC + Gui-A-Gra 1.63 gm/kg p.o. group (VC + G1.63); and (6) VC + Gui-A-Gra 6.5 gm/kg p.o. group (VC + G6.5). Rats were maintained in plastic cases (47 × 18 × 40 cm) (four rats per case) in controlled environment conditions (12-h light-dark cycle, temperature of 20 ± 2 °C, and humidity of 50 ± 10%). Rats had free access to water ad libitum and standard rat chow diet. All experiments were performed according to the Institutional Animal Care and Use Committee (IACUC) of Jeonbuk National University Hospital Laboratory Animal Center (cuh-IACUC-2017-13). After one week of acclimatization, left VC was induced for VC group, VC + G1.63 group, and VC + VC + G6.5 group of rats according to standard protocols [1,2,3]. Rats in CTR, G1.63, and G6.5 groups were sham-operated with an abdominal midline incision only. The midline incision was then sutured. Medication of Gui-A-Gra was started after 4 weeks of VC induction. Gui-A-Gra was diluted in a normal saline and administered through an oral gavage in a daily dose of 1.63 or 6.5 gm/kg body weight to rats of G1.63, G6.5, VC + G1.63, and VC + G6.5 groups for 42 days. Doses of Gui-A-Gra were determined based on the previous study [22]. CTR and VC groups of rats received only normal saline (vehicle) during the medication period. The fertility parameters analysis method was described in the Supplementary Materials (Table S1). At the end of medication for 42 days, rats were anesthetized by intraperitoneal ketamine (100 mg/mL) + 2% xylazine hydrochloride (20 mg/mL) and euthanized by hypothermia and cervical dislocation [1]. Blood samples were collected from the venae cavae of the rats. Serum was separated and stored at −80 °C until biochemical analysis. Reproductive organs were excised and weighed. Half of the left testis tissues were collected in Bouin’s solution for histological analysis and the other half were frozen in liquid nitrogen for biochemical and western blot analyses.

### 4.3. Assessment of Sperm Parameters

Epididymal and vas deference sperm count and sperm motility were evaluated as described previously [45]. In brief, the distal cauda and the vas deference were rapidly washed in PBS to remove excess blood and placed in separate microcentrifuge tubes containing pre-warmed normal saline at 37 °C. Tissues were gently cut with anatomic scissors and sperm were allowed to suspend for 5 min. After incubation, sperm analyses were performed using a counting chamber (SEFI-Medical Instruments Ltd, New York, NY, USA) under a light microscope at ×20 magnification. Sperm count is presented as 10^6^ sperms/mL. Motile spermatozoa were analyzed with the formula of ((mean number of motile spermatozoa/total number of spermatozoa) × 100). Results for sperm motility are expressed as percentages.

### 4.4. Hematoxylin and Eosin (H&E) and Terminal Deoxynucleotidyl Transferase-Mediated (dUTP) Nick-End Labeling (TUNEL) Staining

Testis tissues were fixed in Bouin’s solution, dehydrated, embedded in paraffin wax, and sectioned into 5 μm slices. Testis sections were subjected to hematoxylin and eosin (H&E) staining and TUNEL staining (Dead EndTM Colorimetric TUNEL System for qualitative study; Promega, Madison, WI, USA) as described previously [40]. At least thirty seminiferous tubules (STs) per section were randomly examined under a light microscope (400×). Each section was evaluated using Johnsen’s score [45]. Similarly, at least 30 STs were analyzed for spermatogenic cell density by measuring the diameter of the ST and the thickness of the germinal cell layer using the formula of (diameter of the ST / thickness of the germinal cell layer). For TUNEL assay analysis, dark brown stained cells were counted for at least 30 STs per section under a light microscope (×40 objective). Positive germ cell apoptosis was expressed as TUNEL positive cells/tubules.

### 4.5. Serum Hormonal Assays

Serum levels of testosterone (ab108666, Abcam Cambridge, MA, USA), luteinizing hormone (LH) (E-EL-R0026, Elabscience, Houston, TX, USA), and follicle-stimulating hormone (FSH) (E-EL-R0391, Elabscience, Houston, TX, USA) were measured using enzyme-linked immunosorbent assay (ELISA) per each manufacturer’s protocols.

### 4.6. Measurements of Cytokines

Whole tissue supernatants were prepared as described previously [40]. Testicular levels of interleukin-6 (IL-6) (BMS625 IL-6 rat Elisa kit, Thermo Fisher Scientific, Waltham, MA, USA) and TNF-α (BMS 622 rat TNF-α kit, Thermo Fisher Scientific, Waltham, MA, USA) were measured by enzymatic method using commercial kits as per the manufacturer’s instructions. IL-6 and TNF-α concentrations are expressed per milligram protein.

### 4.7. Measurements of Lipid Peroxidation and Antioxidant Enzymes

Malondialdehyde (MDA) levels in testis tissues were assessed using reagents provided by Northwest Life Science Specialties LLC (Vancouver, WA, USA). Briefly, MDA reaction of a colored complex was measured at an absorbance of 532 nm using a kinetic spectrophotometer (Spectra Max 180, Molecular Devices, Sunnyvale, CA, USA). MDA levels are expressed as μmole of MDA/mg of wet testis tissue. Testicular ROS/RNS levels were quantified using an OxiSelect in Vitro ROS/RNS Assay Kit (STA-347, Cell Biolabs, Inc., San Diego, CA, USA) according to the manufacturer’s protocol. Fluorescence of dichlorofluorescein (DFC) was measured with a SpectraMax Gemini XS fluorimeter (Molecular Devices, Sunnyvale, CA, USA) at excitation/emission wavelengths of 480/530 nm as described previously [1]. Activities of testicular superoxide dismutase (SOD), glutathione peroxidase (GPx), and catalase were detected with commercially available kits (item no. 706002, 703102, and 707002, respectively, Cayman Chemical, Ann Arbor, MI, USA) as per the manufacturer’s instructions. Testicular SOD, GPx, and catalase values are expressed per milligram of protein.

### 4.8. Immunohistochemistry Staining

Paraffin-embedded consecutive testis tissue sections (5 μm in thickness) were deparaffinized with xylene, dehydrated with alcohol, and subjected to heat-mediated 1× target retrieval solution at pH 6.0 (DAKO, Glostrup, Denmark). Endogenous peroxide activity was blocked with peroxidase-blocking solution (DAKO) for 15 min and washed with 1× PBS buffer twice (5 min each). Non-specific bindings of testis section were further blocked with serum block solution for 10 min (DAKO) and incubated with primary antibodies cleaved caspase 3 (1:100, D175, Cell Signaling Technology, Beverly, MA, USA), StAR (1:100, D10H12, Cell Signaling Technology, Beverly, MA, USA), and Grp 78 (1:100, Ab 21, 685, Abcam Cambridge, MA, USA) at 4 °C overnight. Sections were then washed with 1× PBS twice (5 min each) and incubated with a horseradish peroxidase (HRP)-labeled micropolymer conjugated secondary antibody (MP-7451, anti-rabbit IgG; vector Labs, Burlingame, CA, USA) at room temperature for 1 h. Prior to incubation, sections were rinsed with AEC substrate chromogens (SK-4205, ImmPACT AEC Peroxidase substrate; vector Labs, Burlingame, CA, USA). Slides were then rinsed with deionized water for 5 min, counter-stained with hematoxylin, rinsed with distilled water, and mounted with mounting medium (Abcam, Cambridge, MA, USA).

### 4.9. Western Blotting

Extraction of protein from testis tissue was conducted as described previously [1]. Protein levels of pro-caspase-3, cleaved caspase 3, BCL 2 associated X protein (Bax), B-cell lymphoma 2 (Bcl-2), steroidogenic acute regulatory protein (StAR), glutathione peroxidase 4 (GPx 4), ER stress markers, glucose-regulated protein-78 (GRP-78), phosphorylated c-Jun-N-terminal kinase (p-JNK), c-Jun-N-terminal kinase (JNK), phosphorylated inositol-requiring transmembrane kinase/endoribonuclease 1α (p-IRE1α), and inositol-requiring transmembrane kinase/endoribonuclease 1α (IRE1α) in testis tissues were analyzed by Western blotting. Proteins (30–60 μg) were separated by 8% to 12% SDS-polyacrylamide gel electrophoresis and electro-transferred onto PVDF membranes (#1620177, Bio-Rad, Hercules, CA, USA). These membranes were then blocked with 5% non-fat milk for 1 h at 4 °C and incubated with the following primary antibodies: β-actin, pro-caspase-3, cleaved caspase 3, Bcl-2, Bax, and StAR (Cell Signaling Technology, Beverly, MA, USA); GPx 4, GRP-78, and p-IRE1α (Abcam Cambridge, MA USA); IRE1, JNK and p-JNK (Santa Cruz Biotechnology, Dallas, TX, USA) in 5% non-fat milk overnight at 4 °C. Following the incubation, membranes were washed thrice with 0.01% TBS-T (Tris-buffered saline Tween 20, pH 7.2) for 10 min each time and incubated with 1:5000 diluted anti-mouse or anti-rabbit IgG antibody (Cell Signaling Technology, Beverly, MA, USA) at room temperature for 1 h. These membranes were washed with TBST (10 min × 3 times). Signals on membranes were detected using an ECL kit (Amersham Bioscience, Piscataway, NJ, USA) with an ECL visualization system (Vilber Lourmat, France). Band intensities were analyzed using ImageJ software (NIH, Bethesda, MD, USA).

### 4.10. Statistical Analysis

All quantitative data are expressed as mean ± standard error of the mean (SEM). Statistical analyses were performed using SPSS version 22 (IBM, Armonk, NY, USA). One-way analysis of variance (ANOVA) was used to compare groups followed by Tukey’s test for post-hoc analysis. Statistical significance was considered at *p* < 0.05. GraphPad PRISM 6.0 was used for graphical analysis (GraphPad Software, San Diego, CA, USA).

## 5. Conclusions

In summary (Figure 6), our results demonstrate that Gui-A-Gra treatment can enhance normal functions of testis in VC-induced male rats by stimulating testosterone biosynthesis. Gui-A-Gra can ameliorate VC-induced oxidative stress by decreasing lipid peroxidation and the activation of antioxidant enzymes, suppressing inflammation, and downregulating ER stress and mitochondrial medicated germ cell apoptosis pathways, thereby significantly improving reproductive parameters of VC-induced male rats. These findings suggest that Gui-A-Gra may be a promising therapeutic modality for the treatment of varicocele, or as a supplement for ameliorating the reproductive impairment.

## Figures and Tables

**Table 1:** The effect of Gui-A-Gra extract on body weight and reproductive organ weight in varicocele (VC)-induced male SD rats.

Parameters	CTR	G1.63	G6.5	VC	VC + G1.63	VC + G6.5
Body weight (sacrifice; g)	457.41 ± 8.5	450.75 ± 9.90	476.16 ± 9.64	438.91 ± 6.11	448.91 ± 7.97	450.41 ± 9.79
Testis weight (g)	2.15 ± 0.04	2.10 ± 0.06	2.21 ± 0.03	2.01 ± 0.05	2.15 ± 0.03	2.10 ± 0.05
Epididymis weight (g)	0.80 ± 0.02	0.93 ± 0.01	0.89 ± 0.01	0.83 ± 0.01	0.83 ± 0.01	0.82 ± 0.01
Prostate weight (g)	0.96 ± 0.02	0.99 ± 0.02	1.03 ± 0.04	1.01 ± 0.03	0.96 ± 0.03	1.09 ± 0.06
Seminal Vesicle weight (g)	1.94 ± 0.07	2.27 ± 0.08	2.31 ± 0.06	2.10 ± 0.09	2.14 ± 0.07	2.08 ± 0.06
Penis weight (g)	0.38 ± 0.01	0.40 ± 0.01	0.38 ± 0.01	0.35 ± 0.01	0.37 ± 0.01	0.39 ± 0.01
Kidney weight (g)	1.33 ± 0.02	1.40 ± 0.03	1.34 ± 0.01	1.33 ± 0.02	1.38 ± 0.03	1.39 ± 0.04

Data were presented as mean ± S.E.M, *n* = 12. Statistical analyses were performed using one-way ANOVA followed by Tukey’s post hoc test. CTR, control; G1.63, Gui-A-Gra 1.63 gm/kg p.o.; G6.5, Gui-A-Gra 6.5 gm/kg p.o.; VC, varicocele; VC + G1.63, varicocele + Gui-A-Gra 1.63 gm/kg; VC + G 6.5, varicocele + Gui-A-Gra 6.5 gm/kg; G, Gui-A-Gra; p.o., per oral; ANOVA, analysis of variance; SEM: standard error of the mean.

**Table 2:** The effect of Gui-A-Gra extract on sperm count and motility of vas deferens and epididymis.

Parameters	CTR	G1.63	G6.5	VC	VC + G1.63	VC + G6.5
Sperm count (10^6^ /mL)						
Vas deferens	56.65 ± 2.39	50.37 ± 2.82	62.58 ± 1.29	45.91 ± 1.80 **	54.08 ± 1.84	72.41 ± 1.05 ^###^
Epididymis	60.16 ± 2.72	54.79 ± 2.18	64.95 ± 1.25	54.58 ± 2.14	55.54 ± 1.97	77.50 ± 1.43 ^###^
Sperm motility (%)						
Vas deferens	70.93 ± 3.75	62.07 ± 1.95	58.62 ± 1.29	68.82 ± 1.33	78.21 ± 2.40 ^#^	81.37 ± 1.63 ^##^
Epididymis	68.81 ± 3.11	61.64 ± 2.31	58.50 ± 1.71	66.83 ± 1.83	81.80 ± 1.62 ^###^	83.70 ± 1.46 ^###^

Data are presented in mean ± S.E.M, *n* = 12. Statistical analyses were performed using one-way ANOVA followed by Tukey’s post hoc test. ** *p* < 0.01 vs. CTR group, ^#^ *p* < 0.05 vs. VC group, ^##^ *p* < 0.01 vs. VC group, and ^###^ *p* < 0.001 vs. VC group. CTR, control; G1.63, Gui-A-Gra 1.63 gm/kg p.o.; G6.5, Gui-A-Gra 6.5 gm/kg p.o.; VC, varicocele; VC + G1.63, varicocele + Gui-A-Gra 1.63 gm/kg; VC + G6.5, varicocele + Gui-A-Gra 6.5 gm/kg; G, Gui-A-Gra; p.o., per oral; ANOVA, analysis of variance; SEM: standard error of the mean.

## Supplementary Materials

Supplementary materials can be found at https://www.mdpi.com/1422-0067/21/23/9231/s1. Table S1. The fertility rate and pups per female.

## Abbreviations

CTR	Control
Gui-A-Gra	Gryllus bimarculatus powder
G	Gui-A-Gra
G 1.63	Gui-A-Gra 1.63 gm/kg
G 6.5	Gui-A-Gra 6.5 gm/kg
VC	Varicocele
VC + G 1.63	Varicocele + Gui-A-Gra 1.63 gm/kg
VC + G 6.5	Varicocele + Gui-A-Gra 6.5 gm/kg
SD	Sprague-Dawley
ER	Endoplasmic reticulum
Bcl-2	B-cell lymphoma 2
Bax	BCL 2 associated X protein
StAR	Steroidogenic acute regulatory protein
MDA	Malondialdehyde
SOD	Superoxide dismutase
ROS/RNS	Reactive oxygen species/reactive nitrogen species
GPx	Glutathione peroxidase
GPx 4	Glutathione peroxidase 4
LH	Luteinizing hormone
FSH	Follicle stimulating hormone
TNF-α	Tumor necrosis factor-α
IL-6	Interleukin-6
Grp-78	Glucose regulated protein-78
p-JNK	Phosphorylated c-jun-N-terminal kinase
JNK	c-jun-N-terminal kinase
p-IRE1α	Phosphorylated inositol-requiring transmembrane Kinase 1α
IRE1α	Inositol-requiring transmembrane kinase 1α
TUNEL	Terminal deoxynucleotidyl transferase-mediated (dUTP) nick-end labeling
H&E	Hematoxylin and eosin
STs	Seminiferous tubules
PBS	Phosphate buffer saline
DFC	Dichlorofluorescein
GMP	Good manufacturing practice
HACCP	Hazard analysis and critical control point
ANOVA	Analysis of variance
SEM	Standard error of the mean.
p.o.	Per oral
