# Supplementary material for: Gui-A-Gra Attenuates Testicular Dysfunction in Varicocele-Induced Rats via Oxidative Stress, ER Stress and Mitochondrial Apoptosis Pathway
Source: Int J Mol Sci. 2020 Dec 3;21(23):9231. doi: 10.3390/ijms21239231 (PMC7730328; doi:10.3390/ijms21239231)
Supplement: Supplementary file 1 [file ijms-21-09231-s001.pdf]

Supplementary Materials

Table S1 The effect of Gui-A-Gra extract on fertility parameters in VC-induced male SD rats.

Parameters	CTR	G 1.63	G 6.5	VC	VC + G 1.63	VC + G 6.5
Fertility rate (%)	100	100	100	90	100	100
Pups per female	12.80 ± 0.37	12.00 ± 2.44	13.00 ± 0.89	6.60 ± 2.03	12.00 ± 1.04	13.40 ± 1.02#

Data are presented in mean ± S.E.M, n = 5. Statistical analyses were performed using one-way ANOVA followed by Tukey's post hoc test. #*P* < 0.05 vs. VC group. CTR, control; G 1.63, Gui-A-Gra 1.63 gm/kg p.o; G 6.5, Gui-A-Gra 6.5 gm/kg p.o; VC, varicocele; VC + G 1.63, varicocele + Gui-A-Gra 1.63 gm/kg; VC + G 6.5, varicocele + Gui-A-Gra 6.5 gm/kg; G, Gui-A-Gra; p.o., per oral; ANOVA, analysis of variance; SEM: standard error of the mean.

Assessment of fertility capacity

The fertility parameters were analyzed as previously described [1].Thirty female rats were used to determine fertility rate and pups per female after natural mating. Each mating pair was kept in single case after 4 weeks of medication. After 2 weeks, female rats were separated from male rats and kept in a separate singled case. Male rats were proven fertility by producing offspring.

1. Karna KK, Choi BR, You JH, Shin YS, Soni KK, Cui WS et al. Cross-talk between ER stress and mitochondrial pathway mediated adriamycin-induced testicular toxicity and DA-9401 modulate adriamycin-induced apoptosis in Sprague-Dawley rats. Cancer cell international 2019;19:85.
